# Supplementary material for: Closo-Carboranyl- and Metallacarboranyl [1,2,3]triazolyl-Decorated Lapatinib-Scaffold for Cancer Therapy Combining Tyrosine Kinase Inhibition and Boron Neutron Capture Therapy
Source: Cells. 2020 Jun 5;9(6):1408. doi: 10.3390/cells9061408 (PMC7349914; doi:10.3390/cells9061408)
Supplement: Supplementary file 1 [file cells-09-01408-s001.pdf]

Closo-carboranyl- and Metallacarboranyl[1,2,3]triazolyl-decorated Lapatinib-scaffold for Cancer Therapy Combining Tyrosine Kinase Inhibition and Boron Neutron Capture Therapy

Marcos Couto, Catalina Alamón, María Fernanda García, Mariángeles Kovacs, Emiliano Trias, Susana Nievas, Emiliano Pozzi, Paula Curotto, Silvia Thorp, María Alejandra Dagrosa, Francesc Teixidor, Clara Viñas and Hugo Cerecetto

Supplementary Material

Spectra developed compounds ……………………………pages 2–15
Figure S1 …………………………………page 16
Figure S2 …………………………………page 17

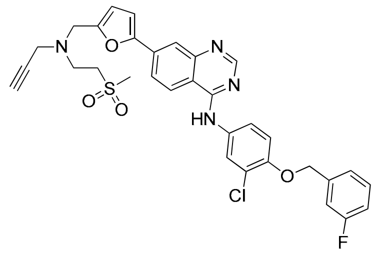

^{1}H-NMR

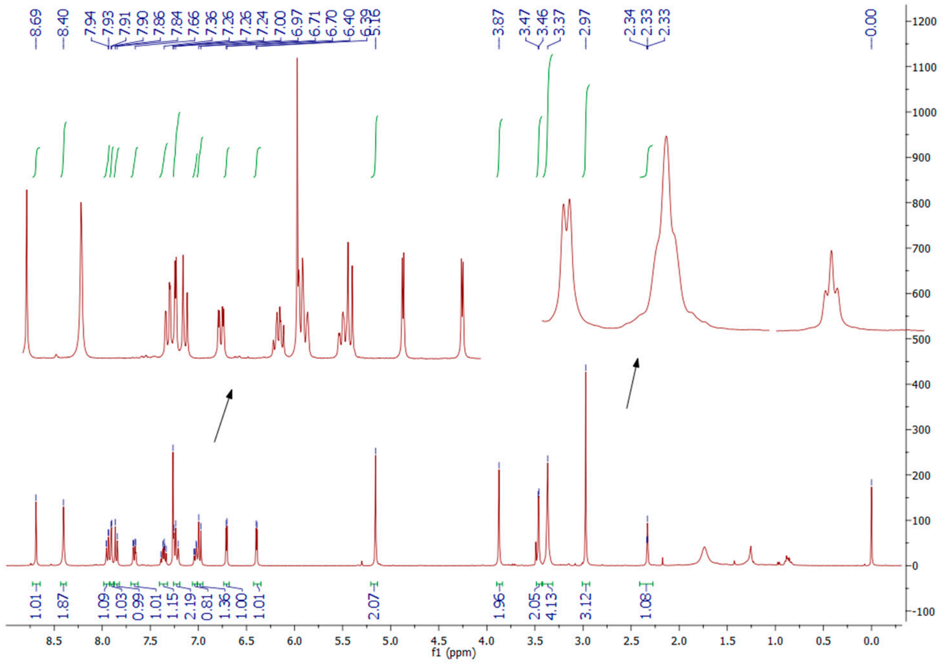

^{13}C-NMR

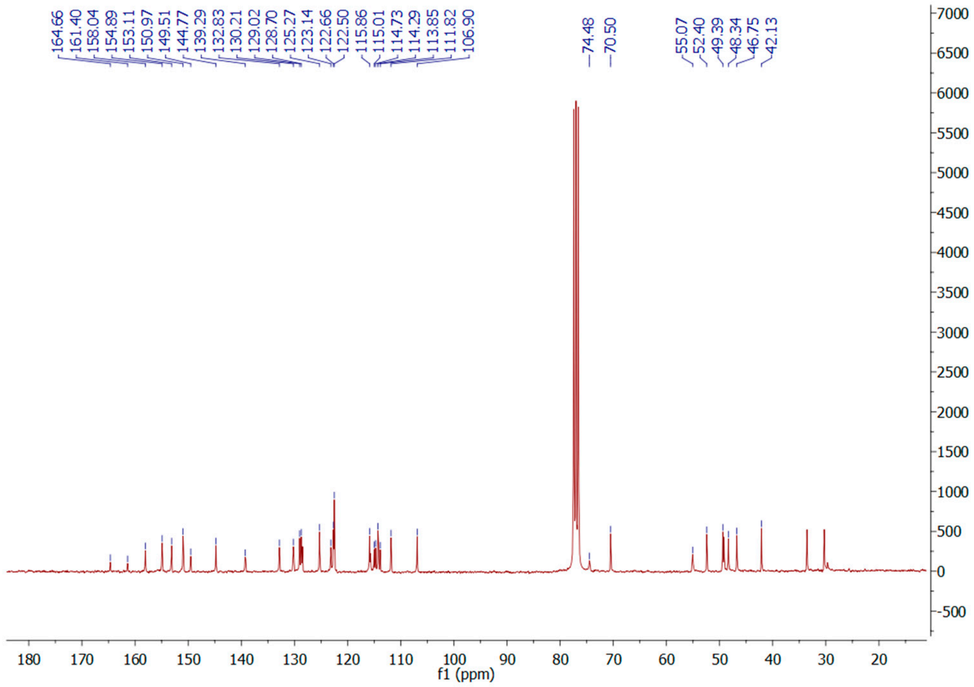

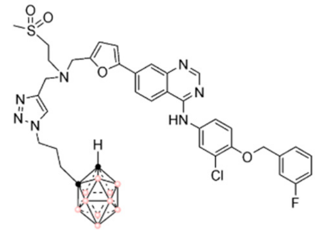

$^{1}H\{^{11}B\}$-NMR

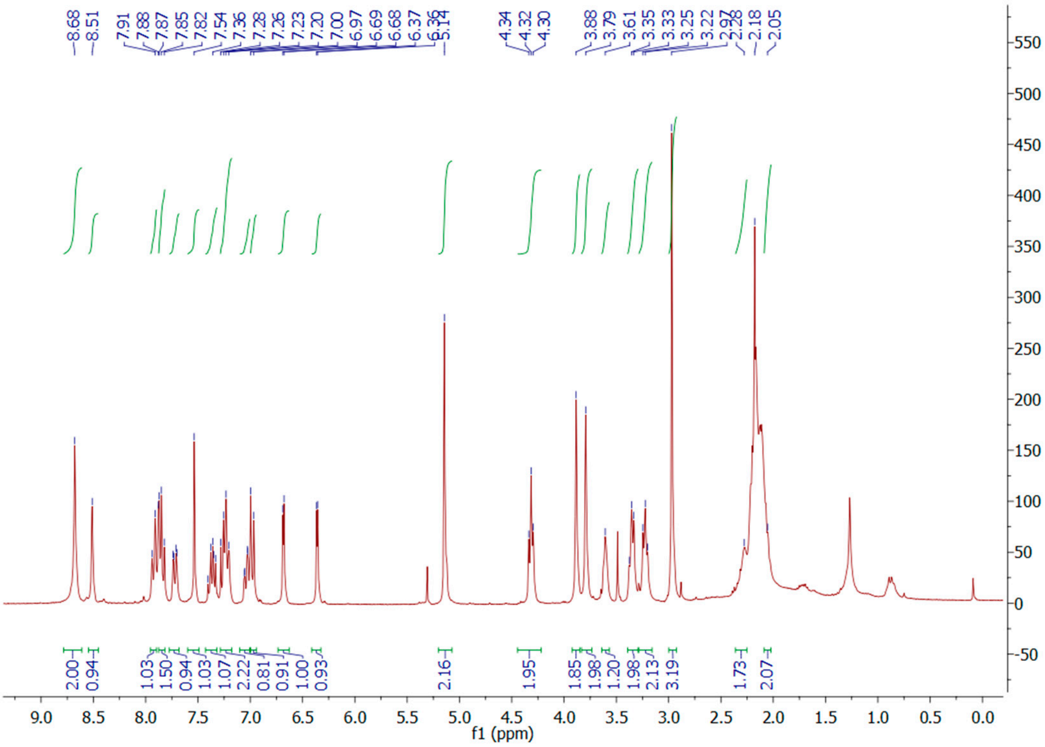

^{1}H-NMR

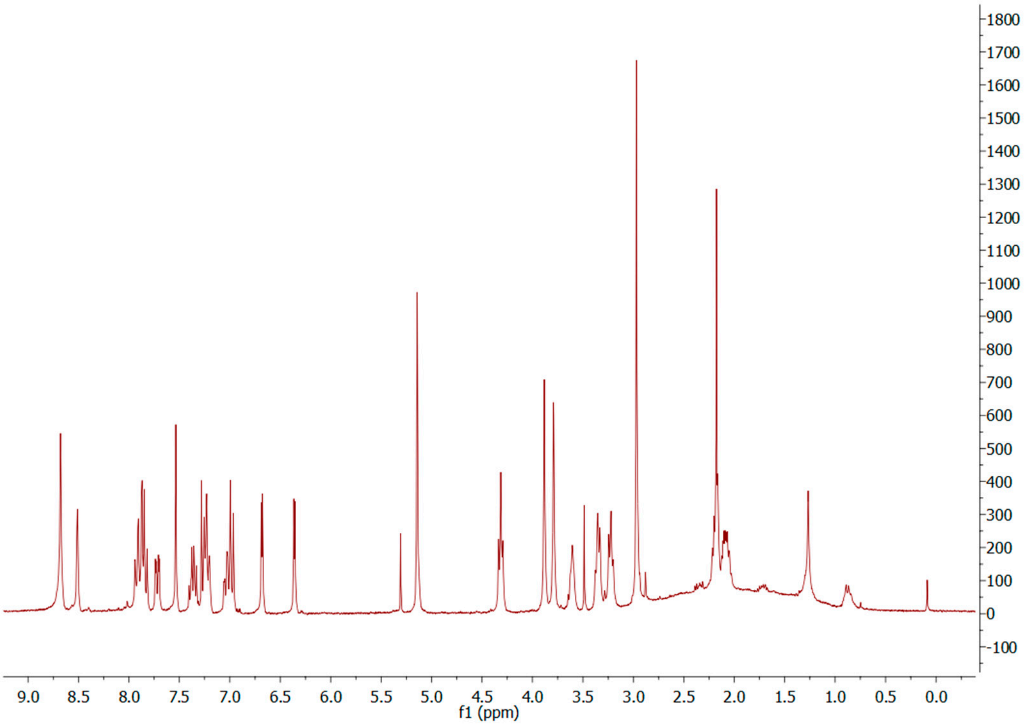

^{13}C-NMR

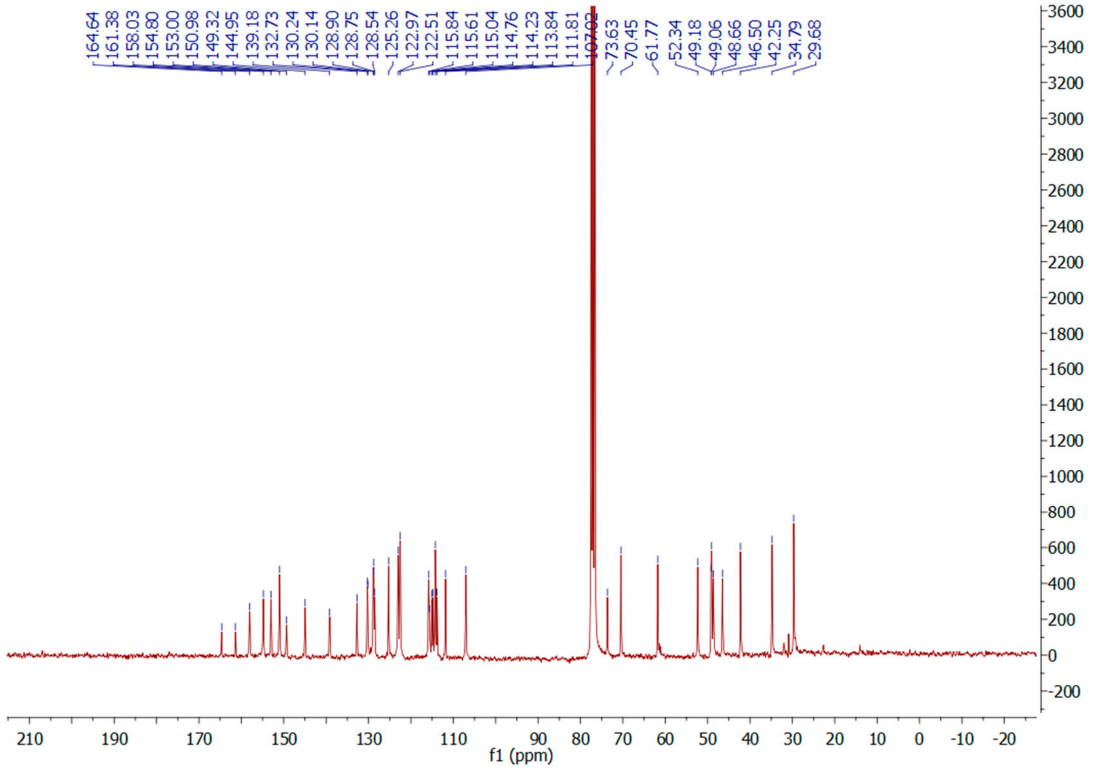
164.64
161.38
158.03
154.80
153.00
150.98
149.32
144.95
139.18
132.73
130.24
130.14
128.90
128.75
128.54
125.26
122.97
122.51
115.84
115.61
115.04
114.76
114.23
113.84
111.81
73.63
70.45
61.77
52.34
49.18
49.06
48.66
46.50
42.25
34.79
29.68
f1 (ppm)

$^{11}B\{^{1}H\}$-NMR

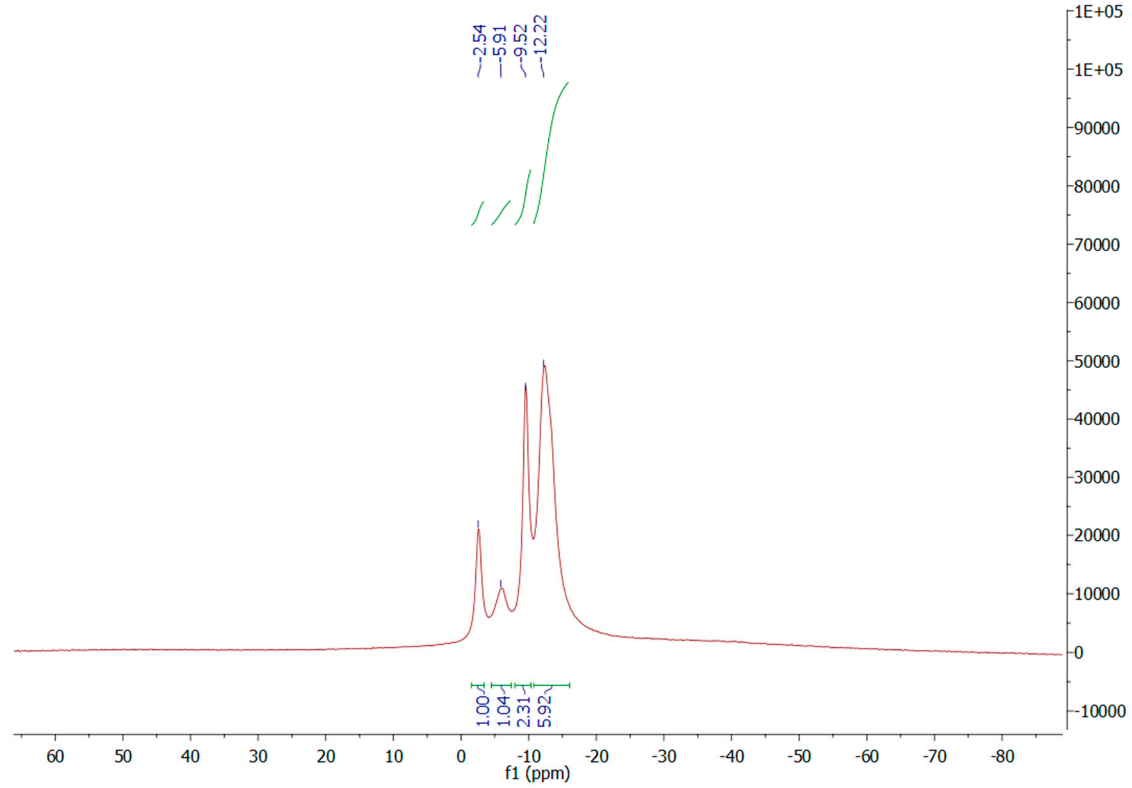
-2.54
-5.91
-9.52
-12.22
1.00
1.04
2.31
5.92
f1 (ppm)

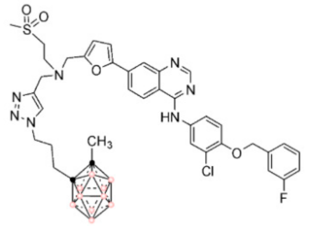

^{1}H-NMR

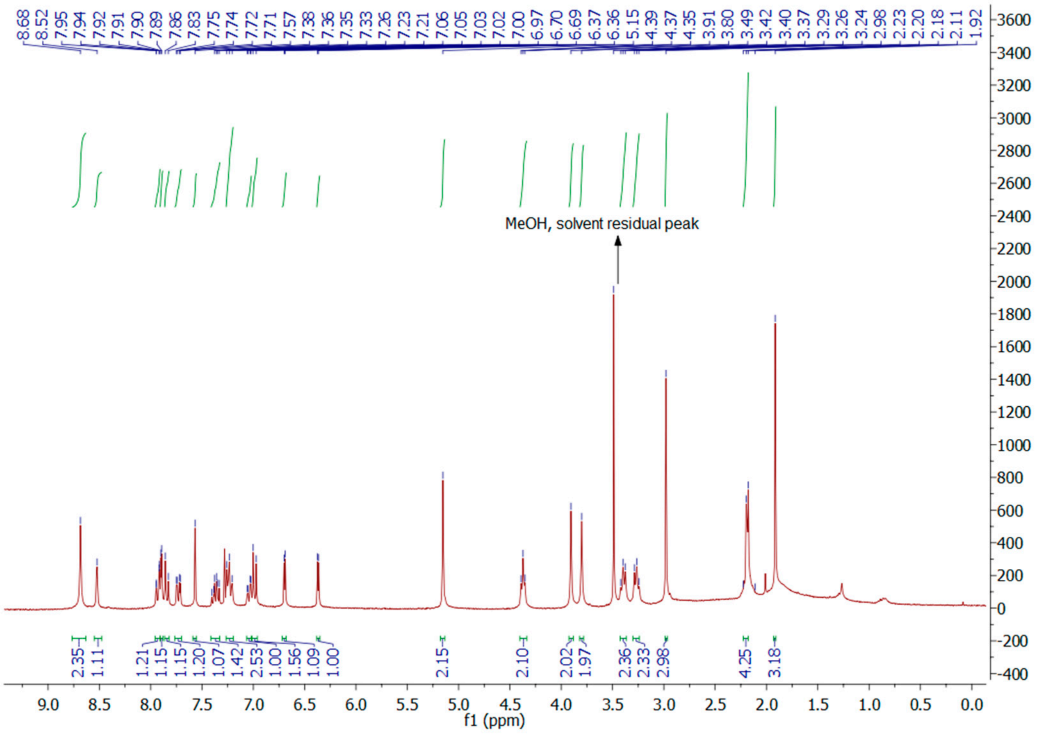

^{13}C-NMR

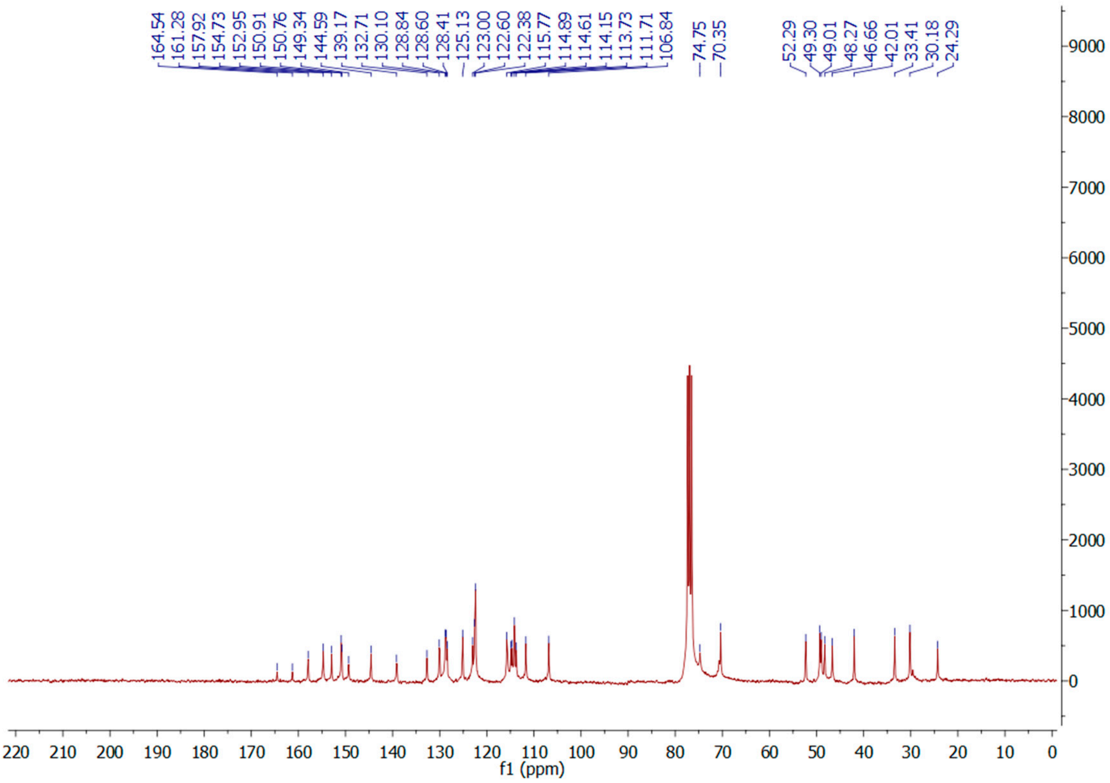

$^{1}H\{^{11}B\}$-NMR

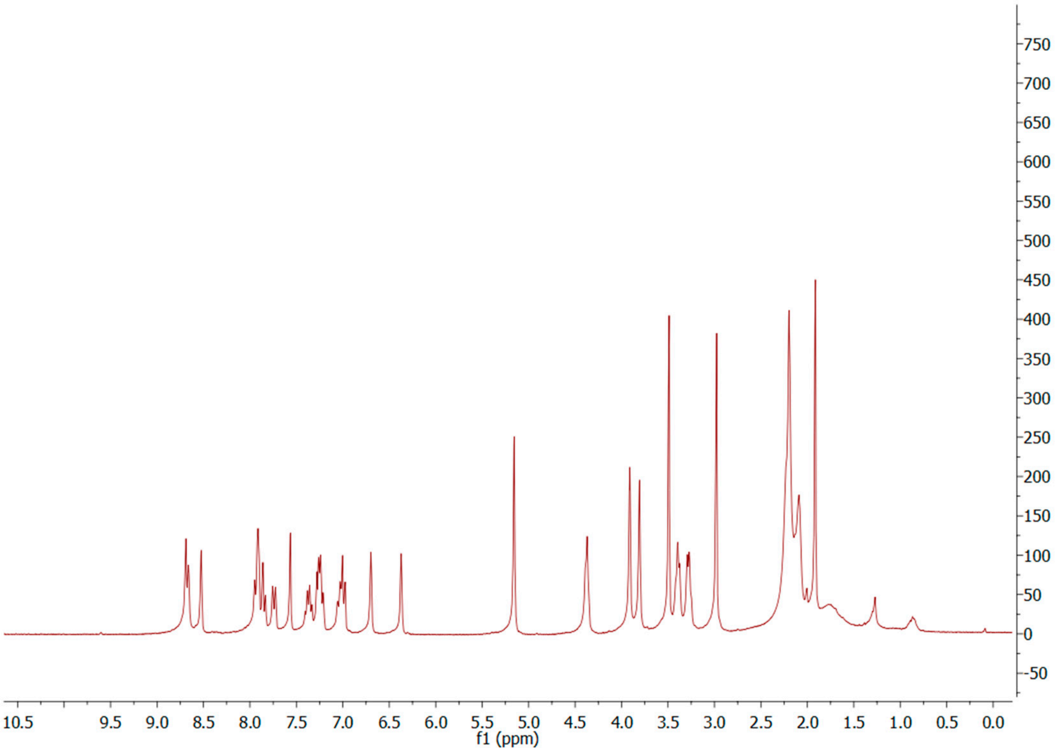

$^{11}B\{^{1}H\}$-NMR

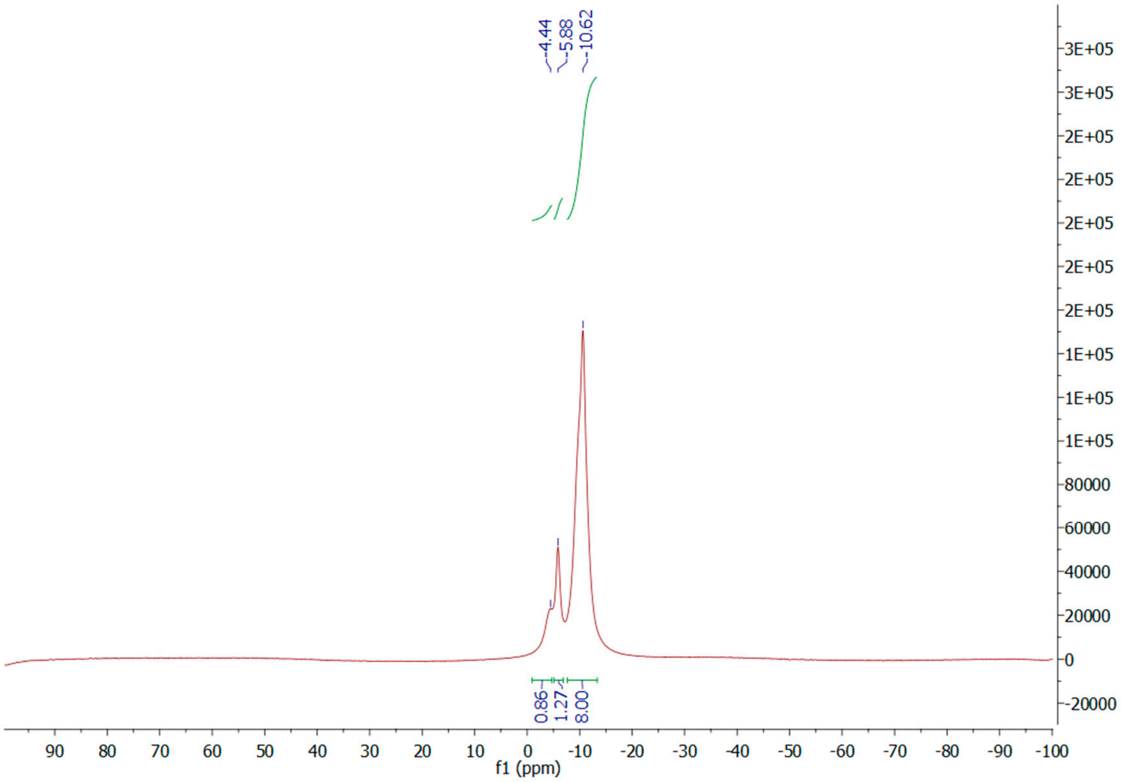

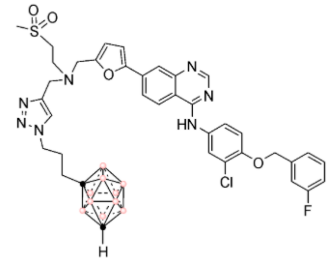

$^{1}H\{^{11}B\}$-NMR

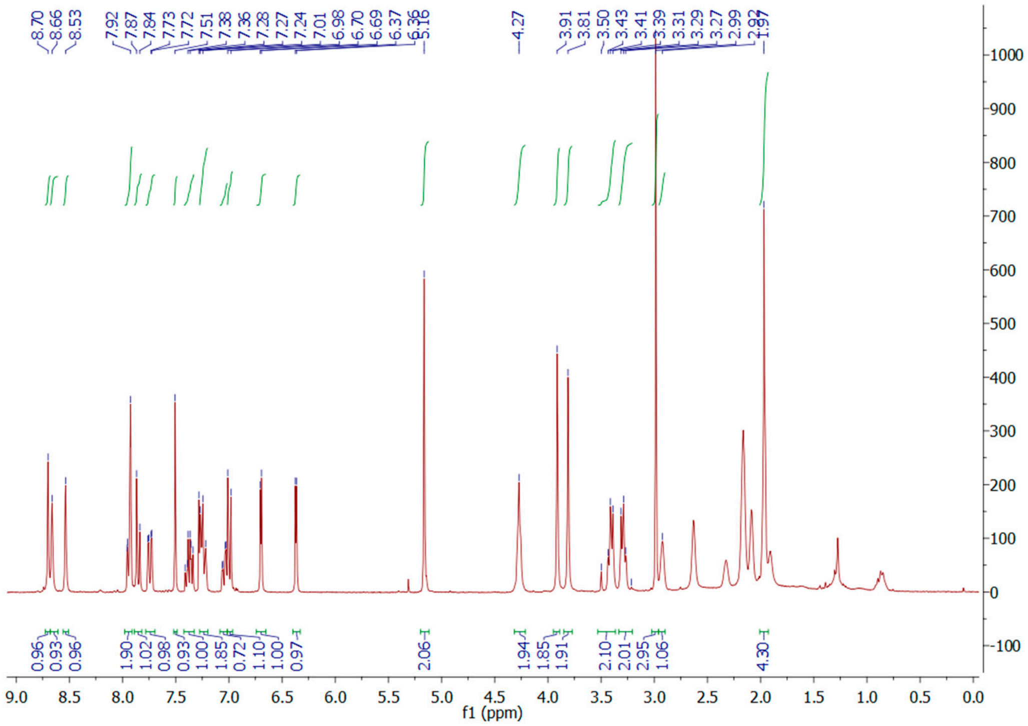

^{1}H-NMR

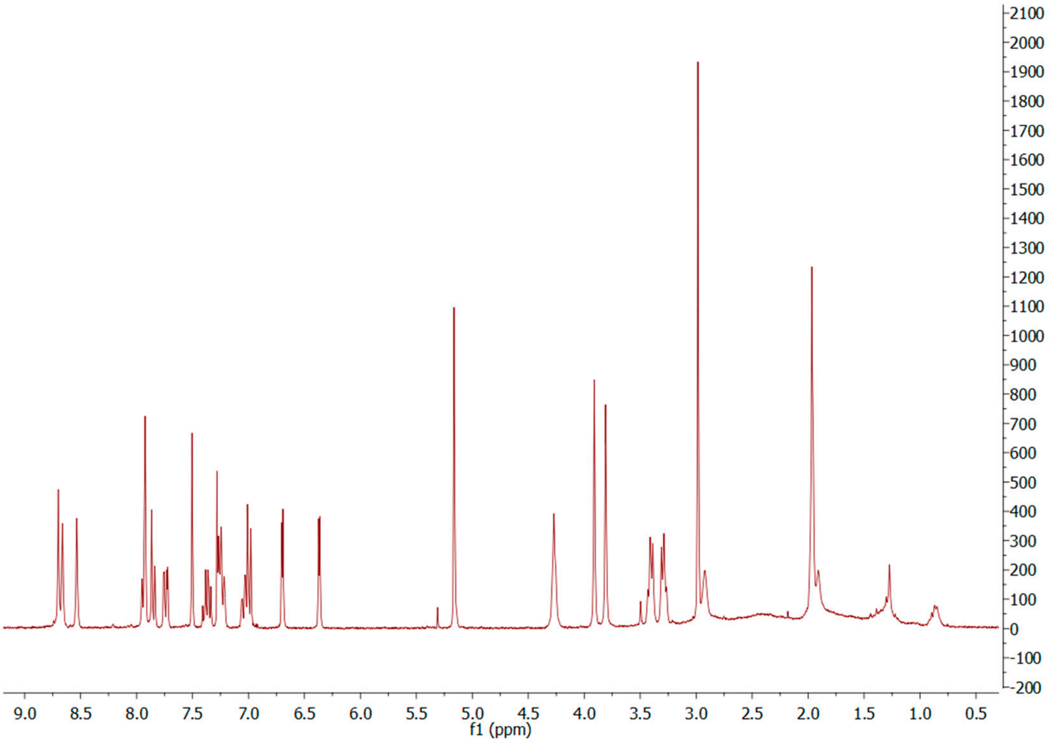

^{13}C-NMR

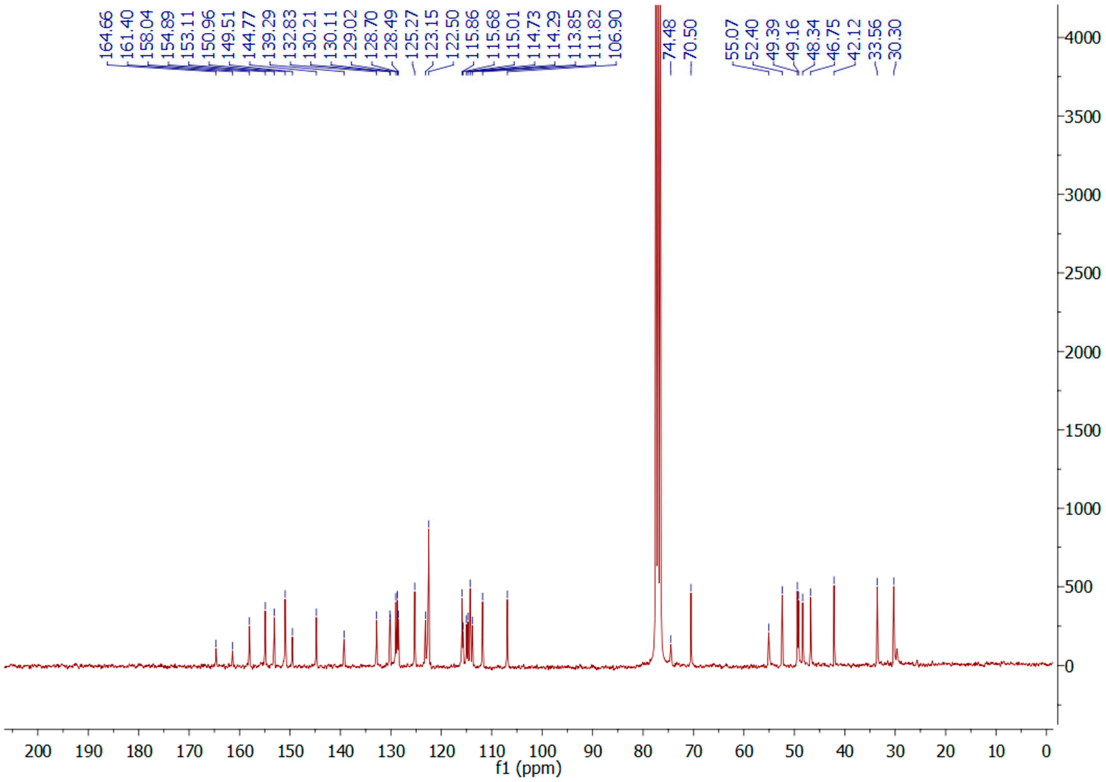

^{11}B{^{1}H}-NMR

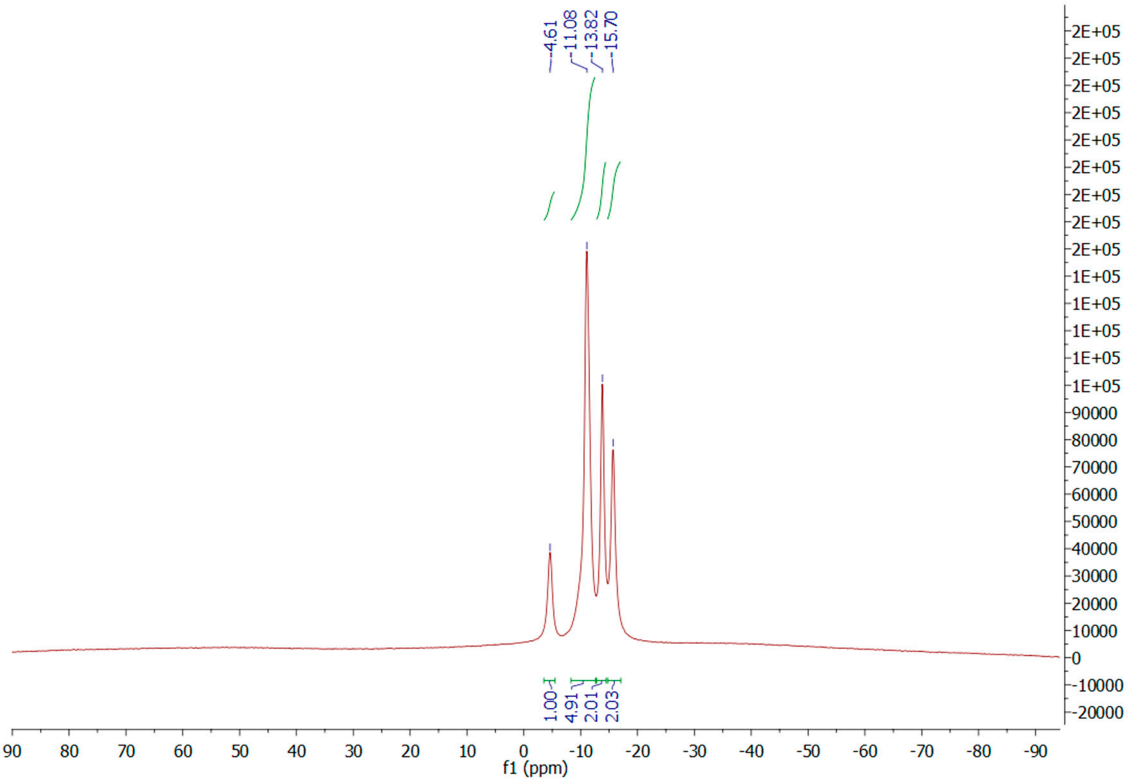

^{11}B-NMR

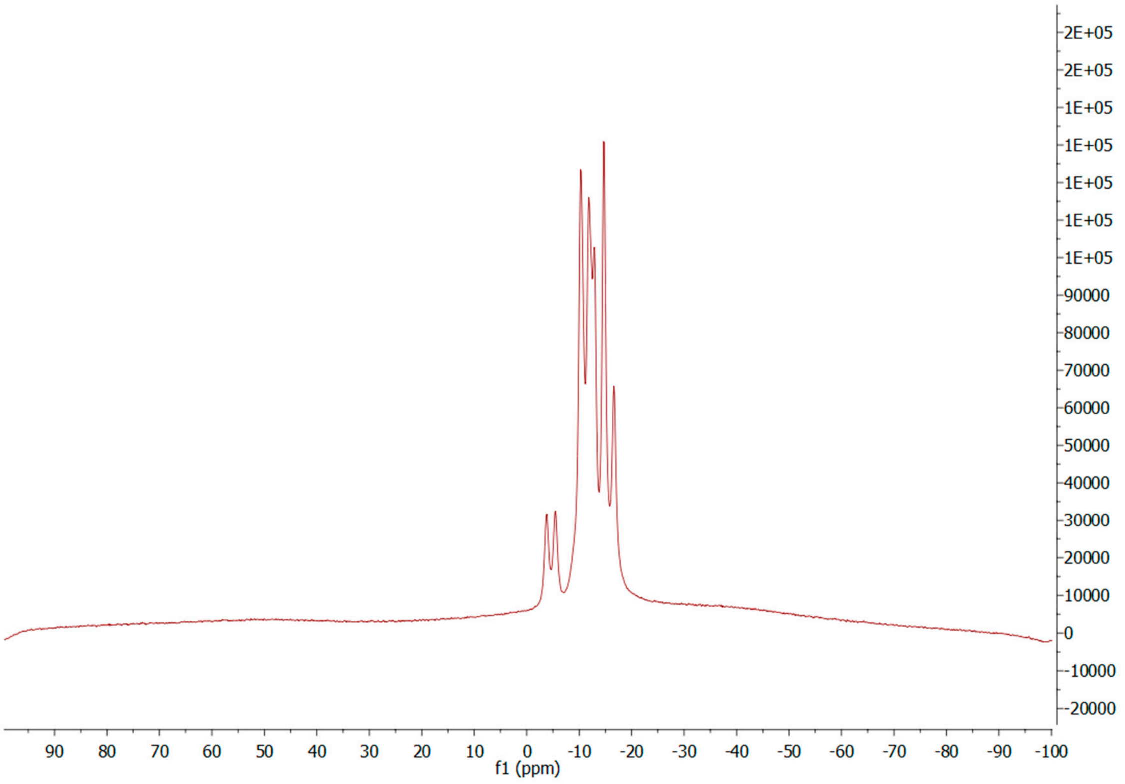
2E+05
2E+05
1E+05
1E+05
1E+05
1E+05
1E+05
90000
80000
70000
60000
50000
40000
30000
20000
10000
0
-10000
-20000
90 80 70 60 50 40 30 20 10 0 -10 -20 -30 -40 -50 -60 -70 -80 -90 -100
f1 (ppm)

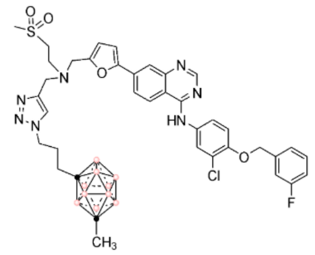

$^{1}H\{^{11}B\}$-NMR

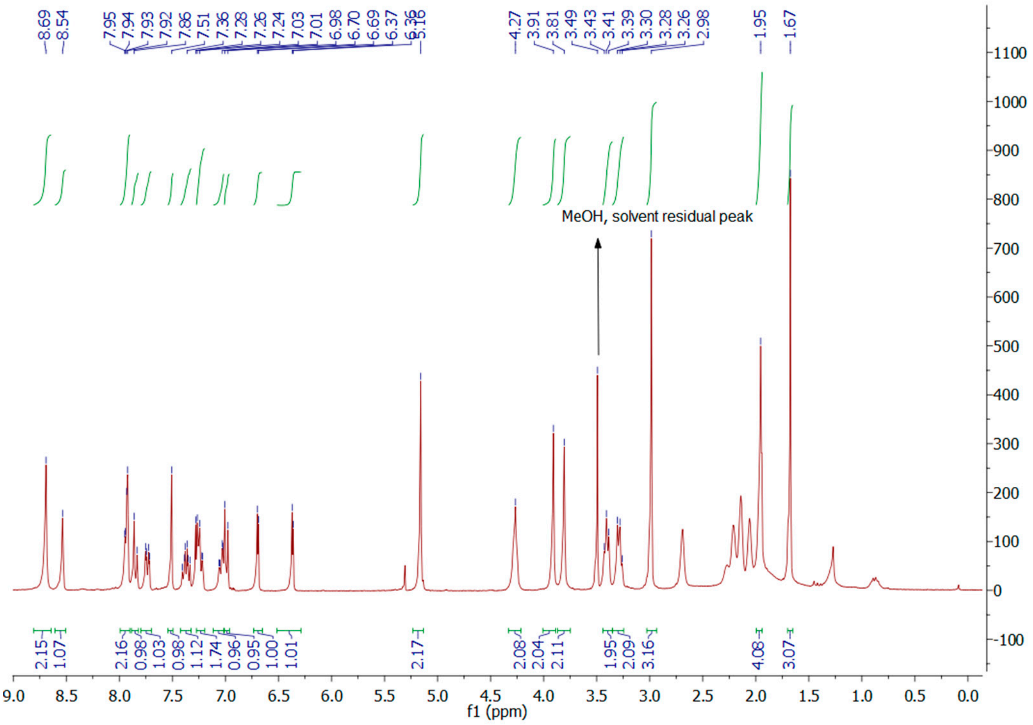

^{1}H-NMR

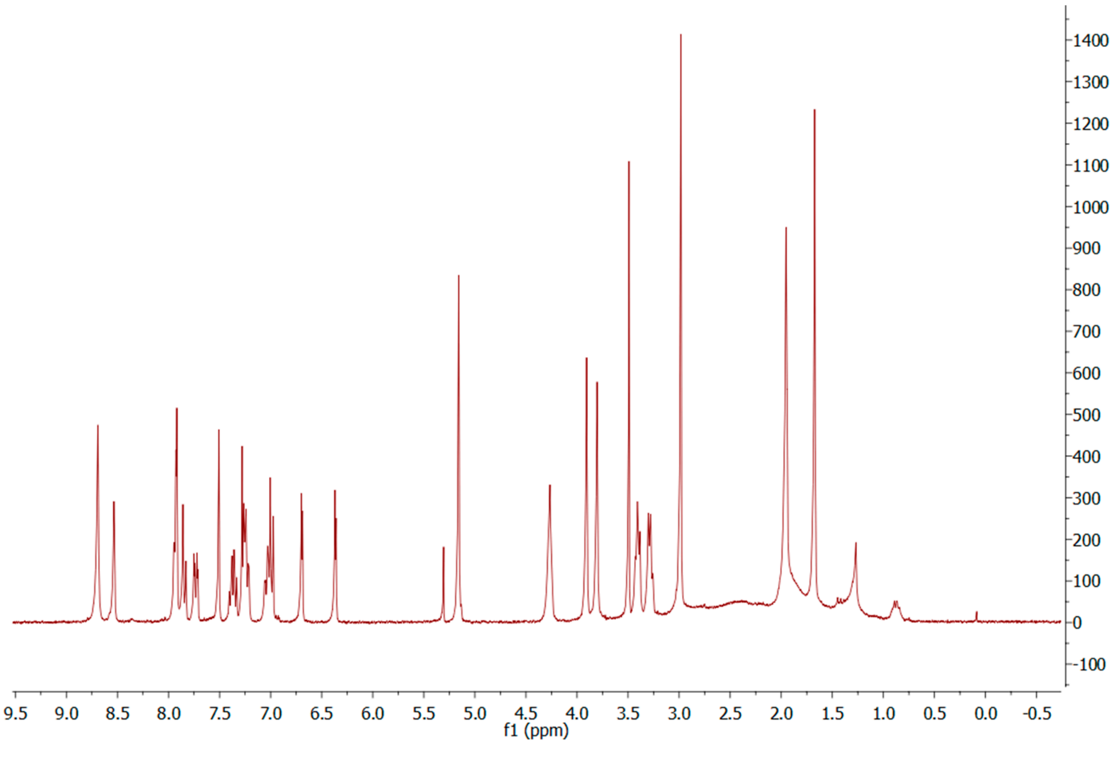

^{13}C-NMR

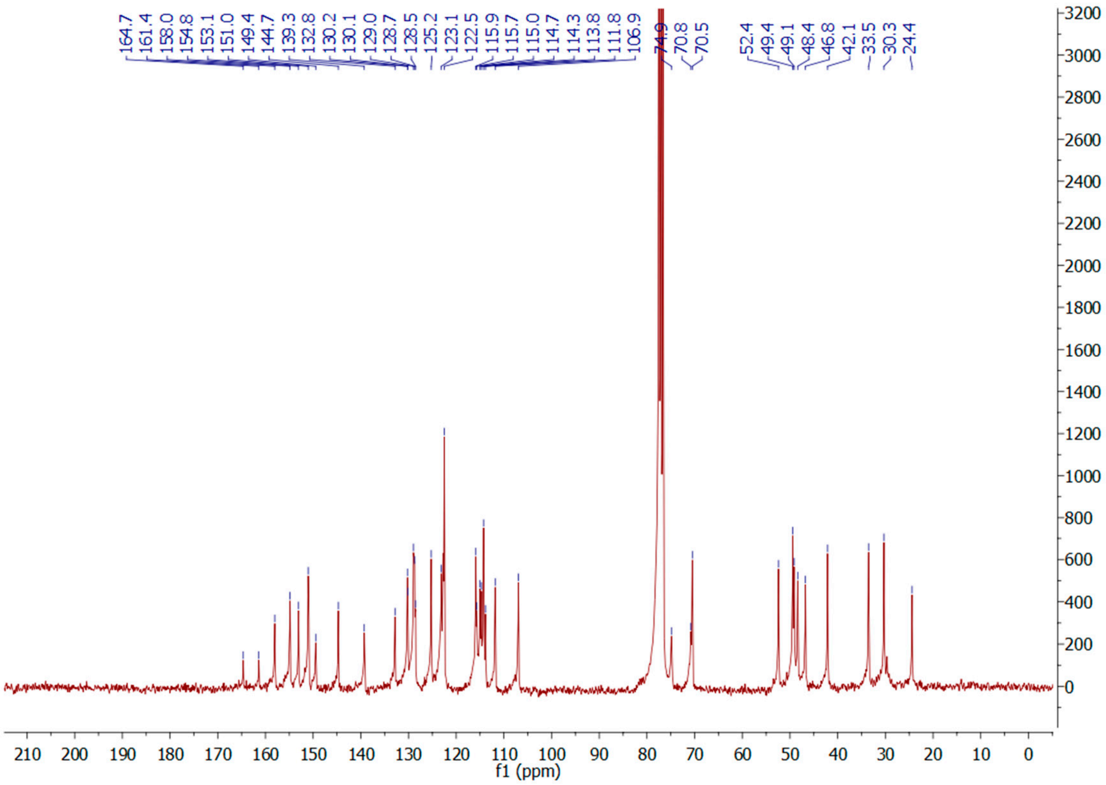
164.7
161.4
158.0
154.8
153.1
151.0
149.4
144.7
139.3
132.8
130.2
130.1
129.0
128.7
128.5
125.2
123.1
122.5
115.9
115.7
115.0
114.7
114.3
113.8
111.8
106.9
74.9
70.8
70.5
52.4
49.4
49.1
48.4
46.8
42.1
33.5
30.3
24.4
f1 (ppm)

$^{11}B\{^{1}H\}$-NMR

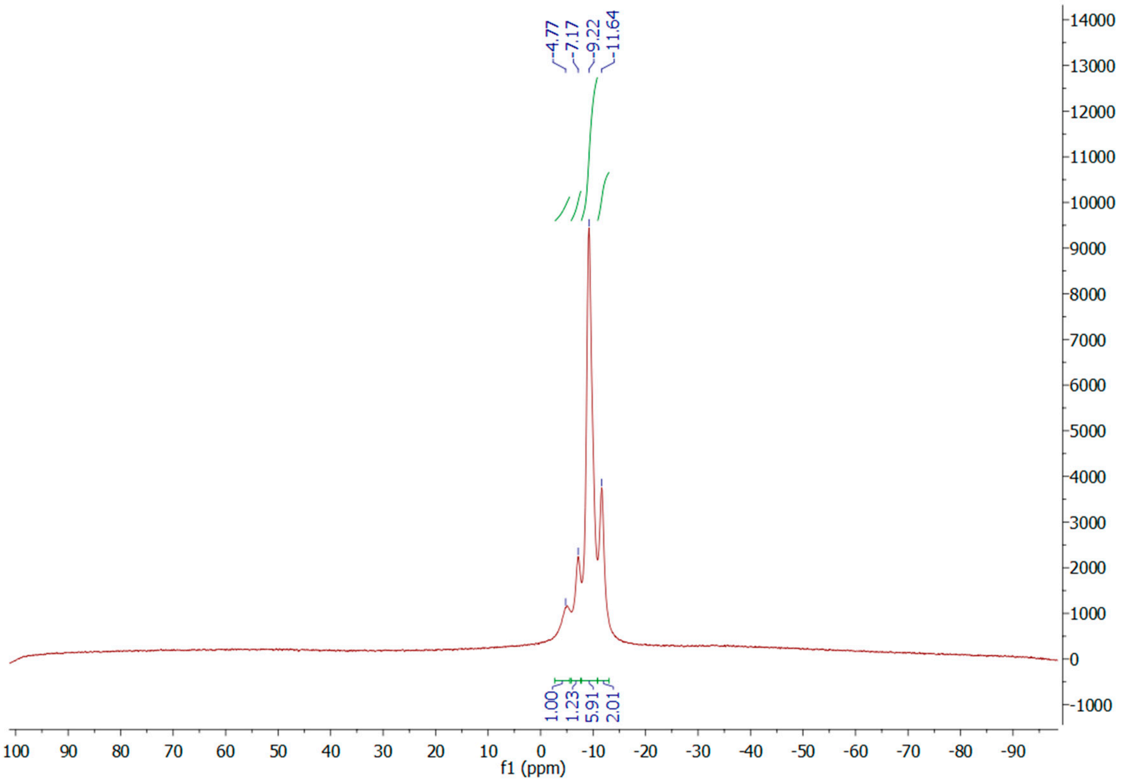
-4.77
-7.17
-9.22
-11.64
1.00
1.23
5.91
2.01
f1 (ppm)

^{11}B-NMR

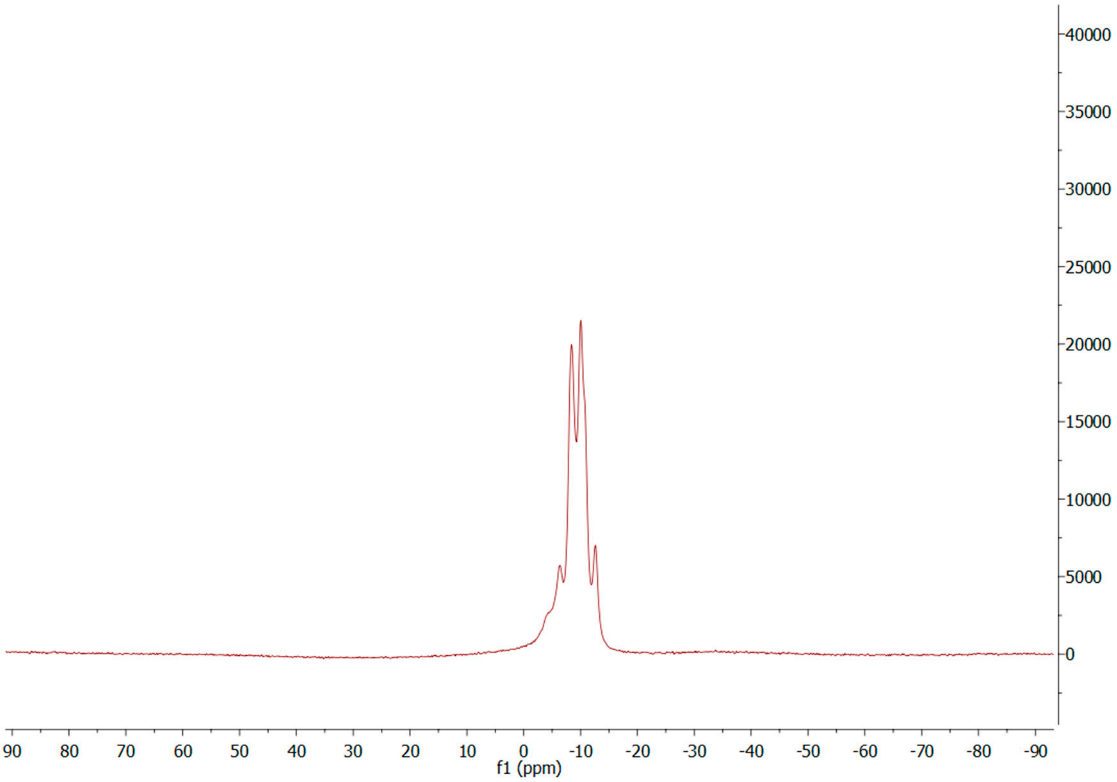

40000
35000
30000
25000
20000
15000
10000
5000
0
90 80 70 60 50 40 30 20 10 0 -10 -20 -30 -40 -50 -60 -70 -80 -90
f1 (ppm)

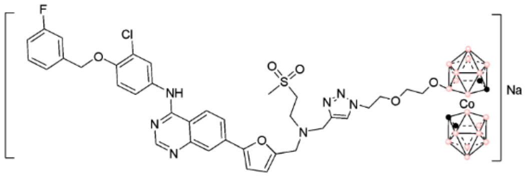

^{1}H{^{11}B}-NMR

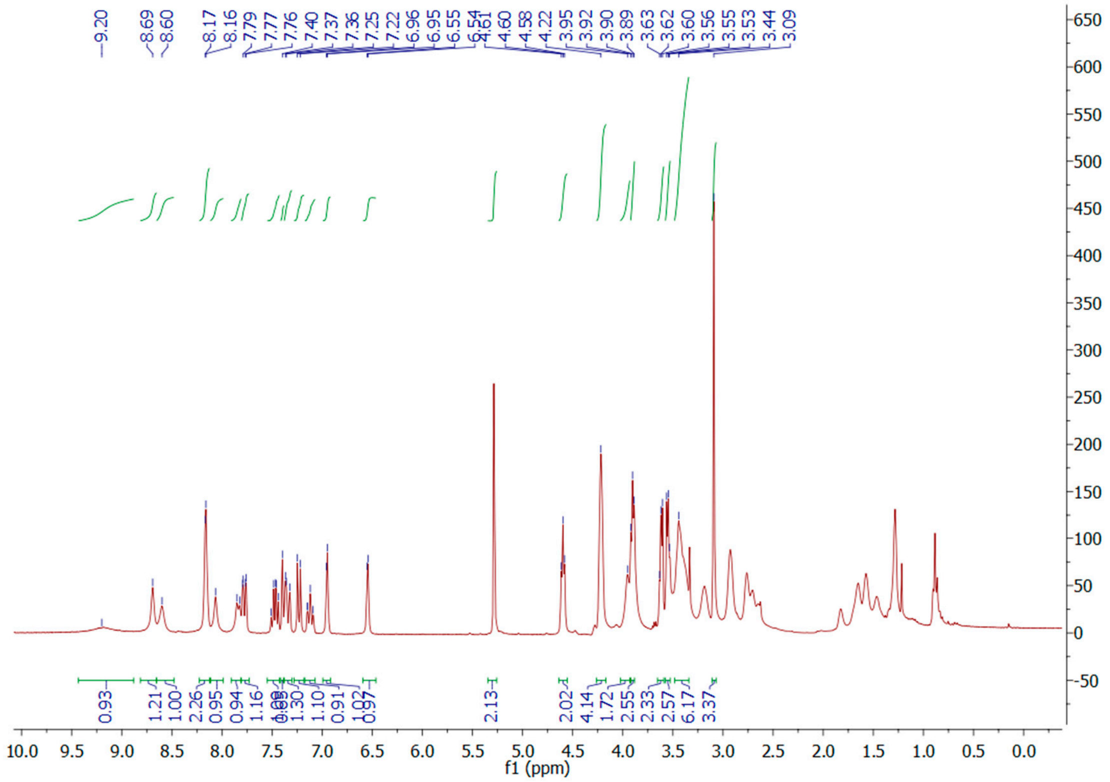

^{13}C-NMR

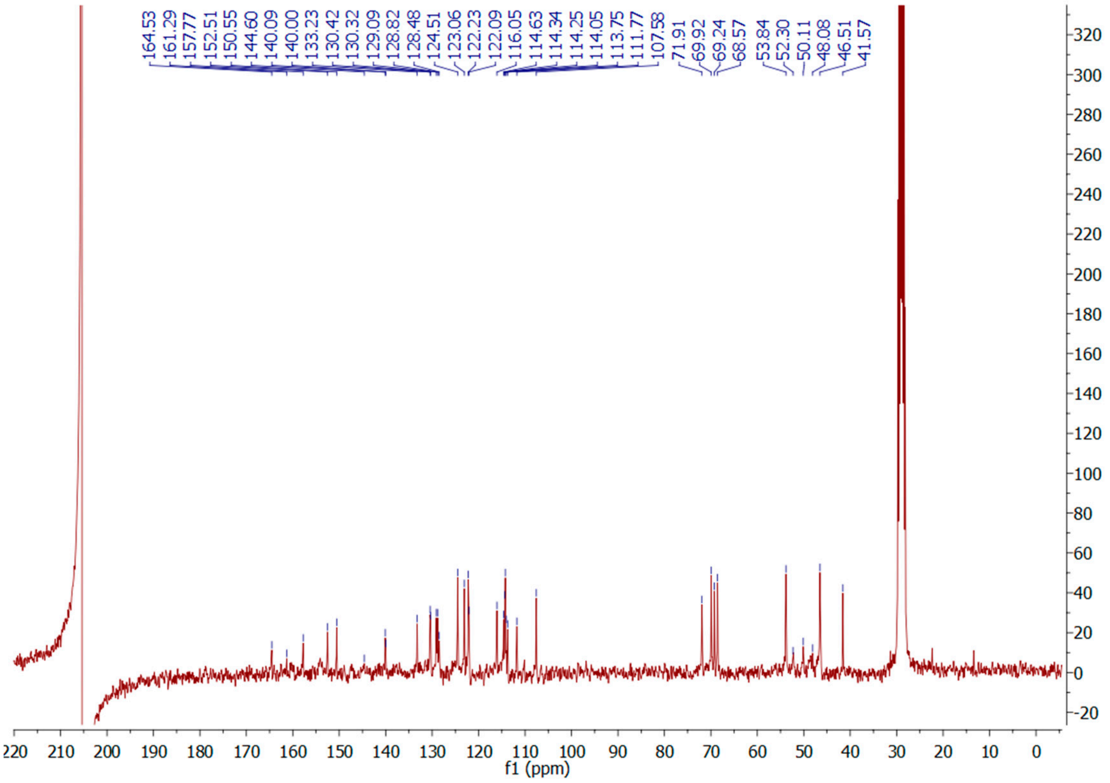

$^{11}B\{^1H\}$-NMR

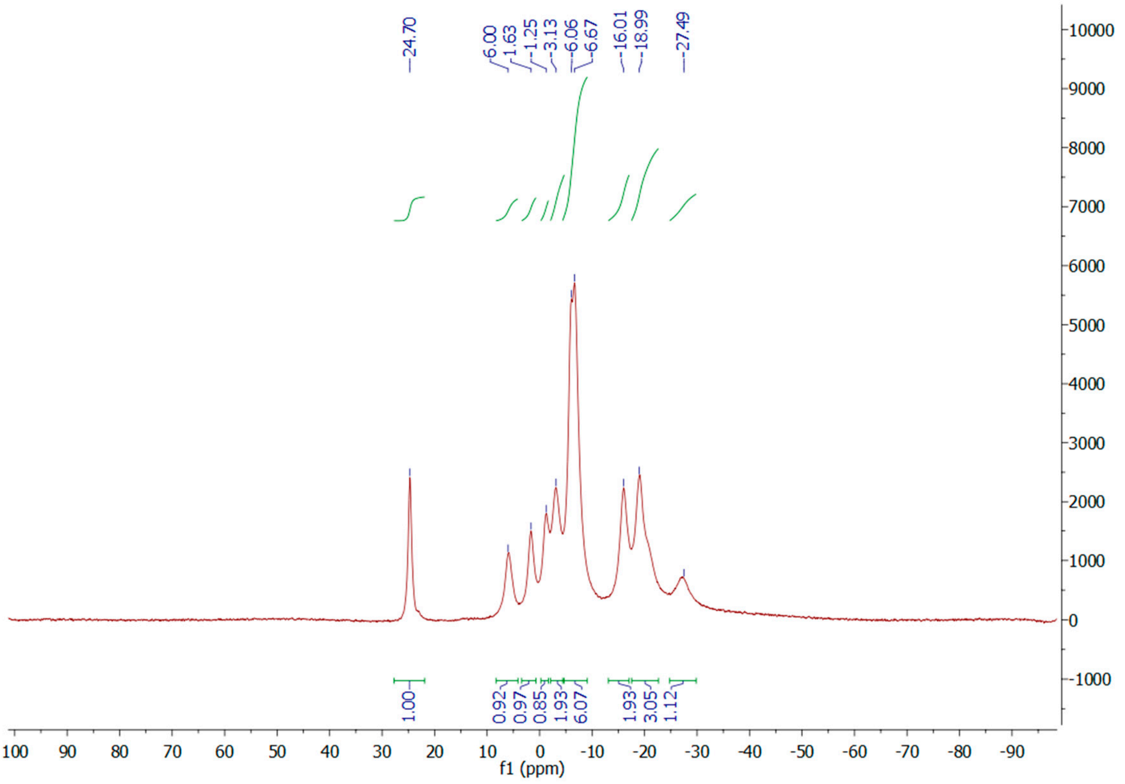

MALDI-TOF-MS

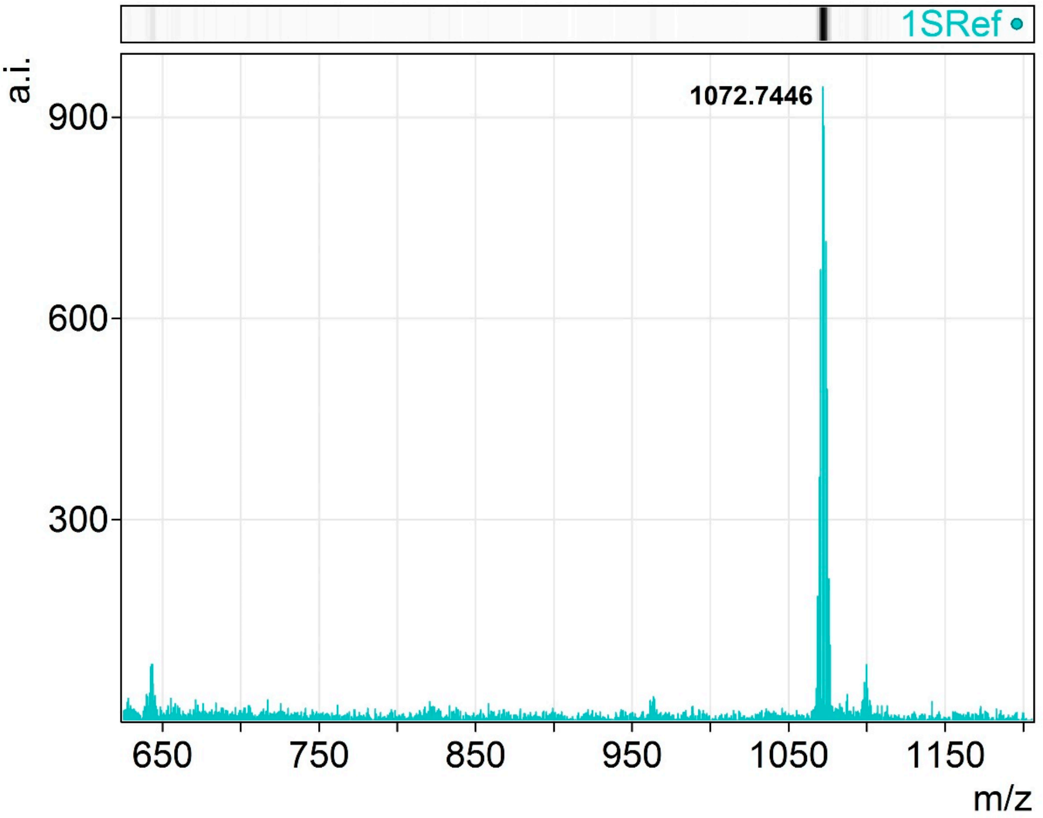

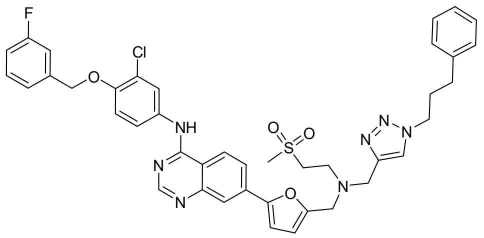

^{1}H-NMR

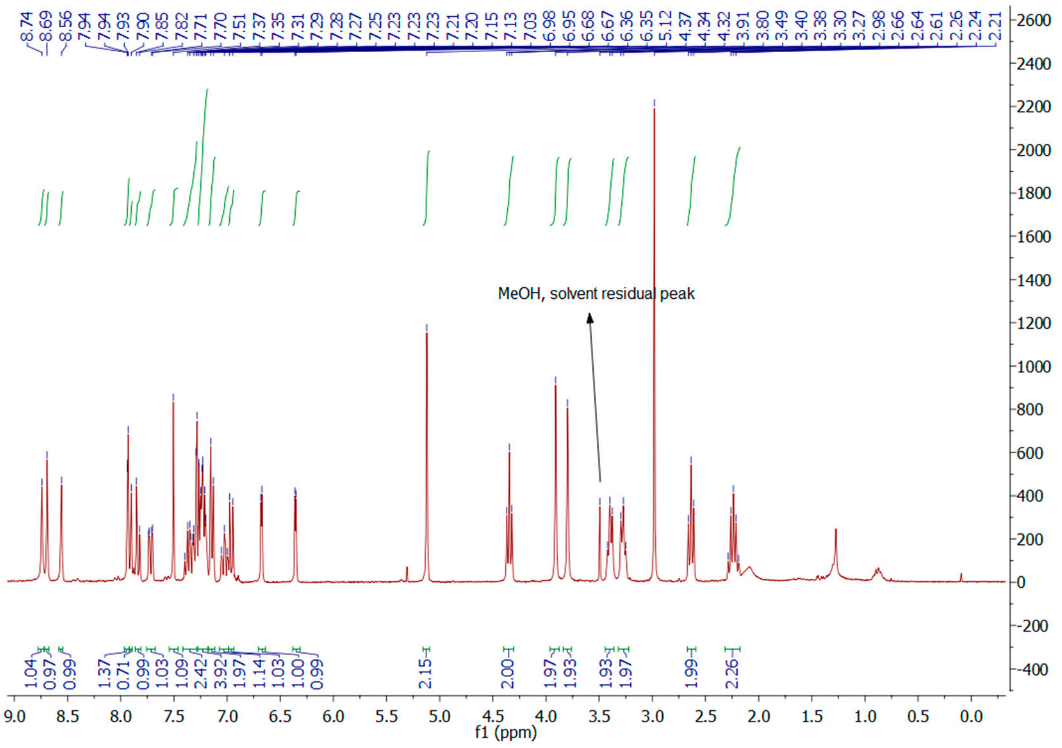

^{13}C-NMR

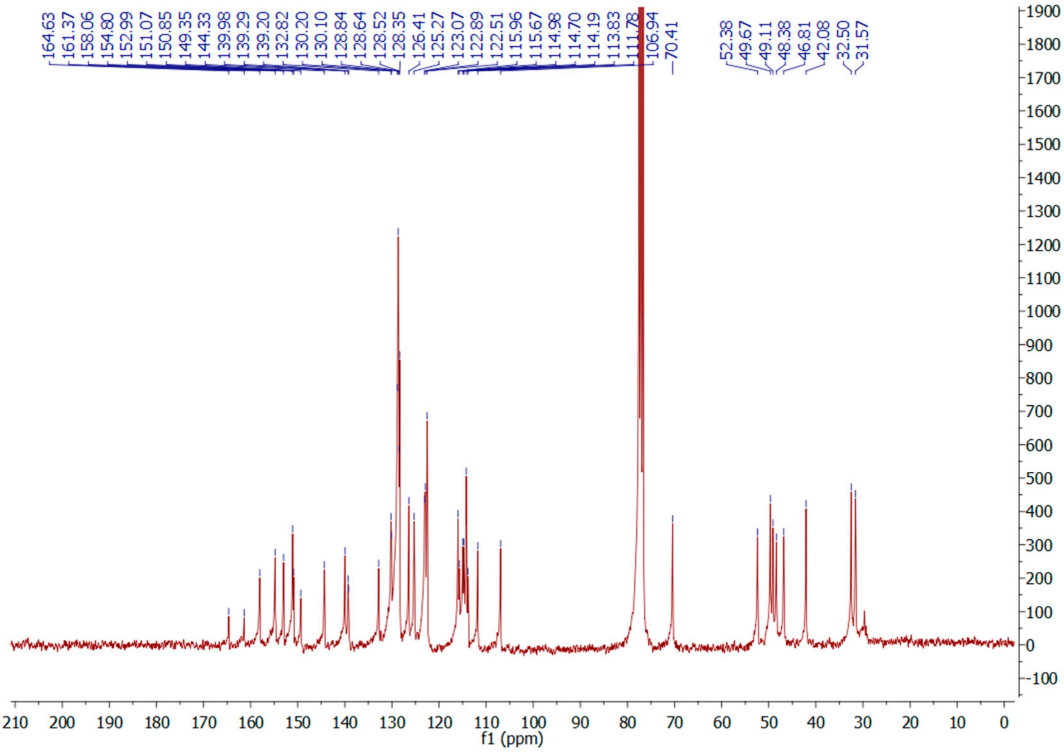

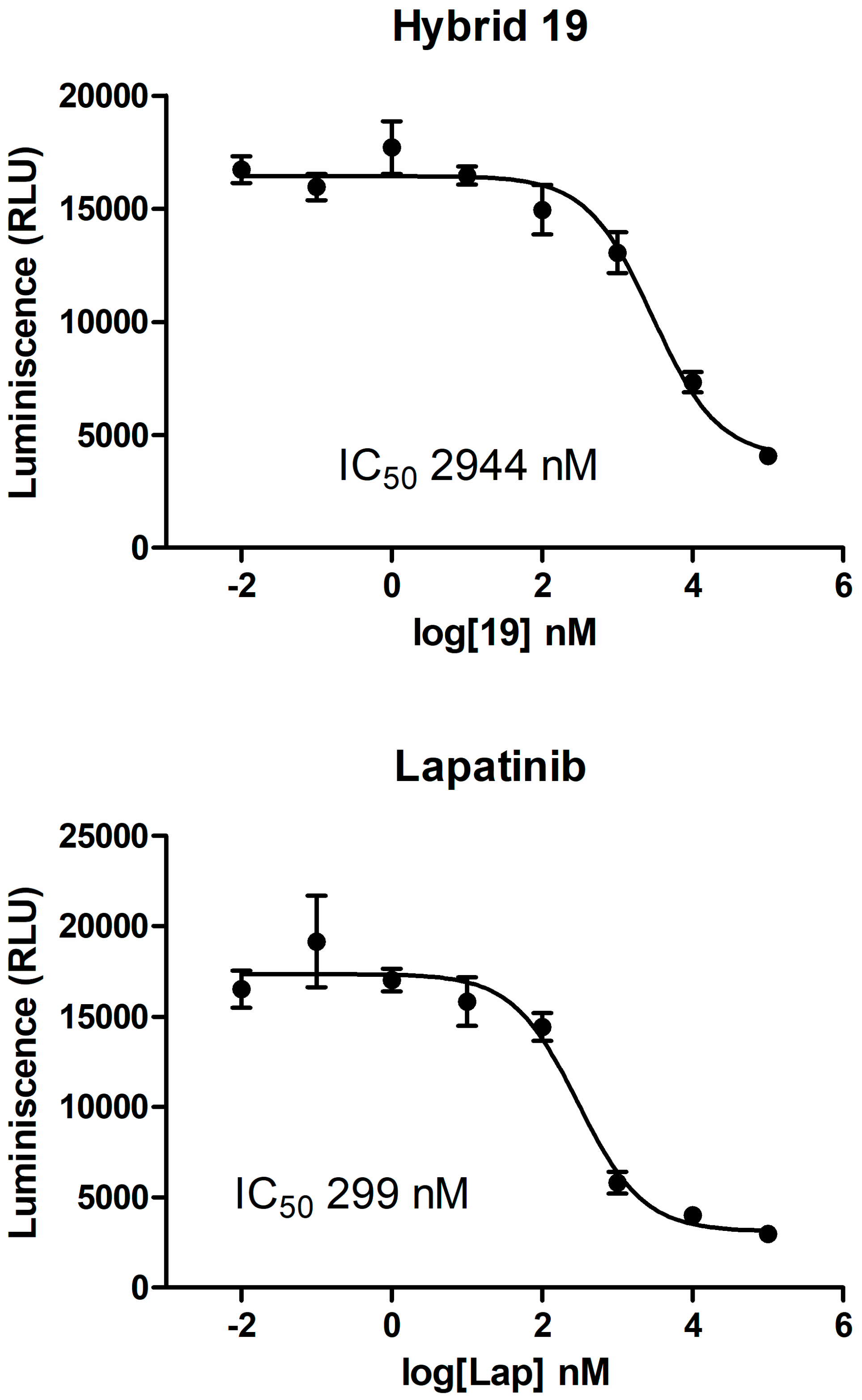

Figure S1. EGFR inhibition. Dose-response curves for hybrid, **19** and **Lap**.

Lapatinib

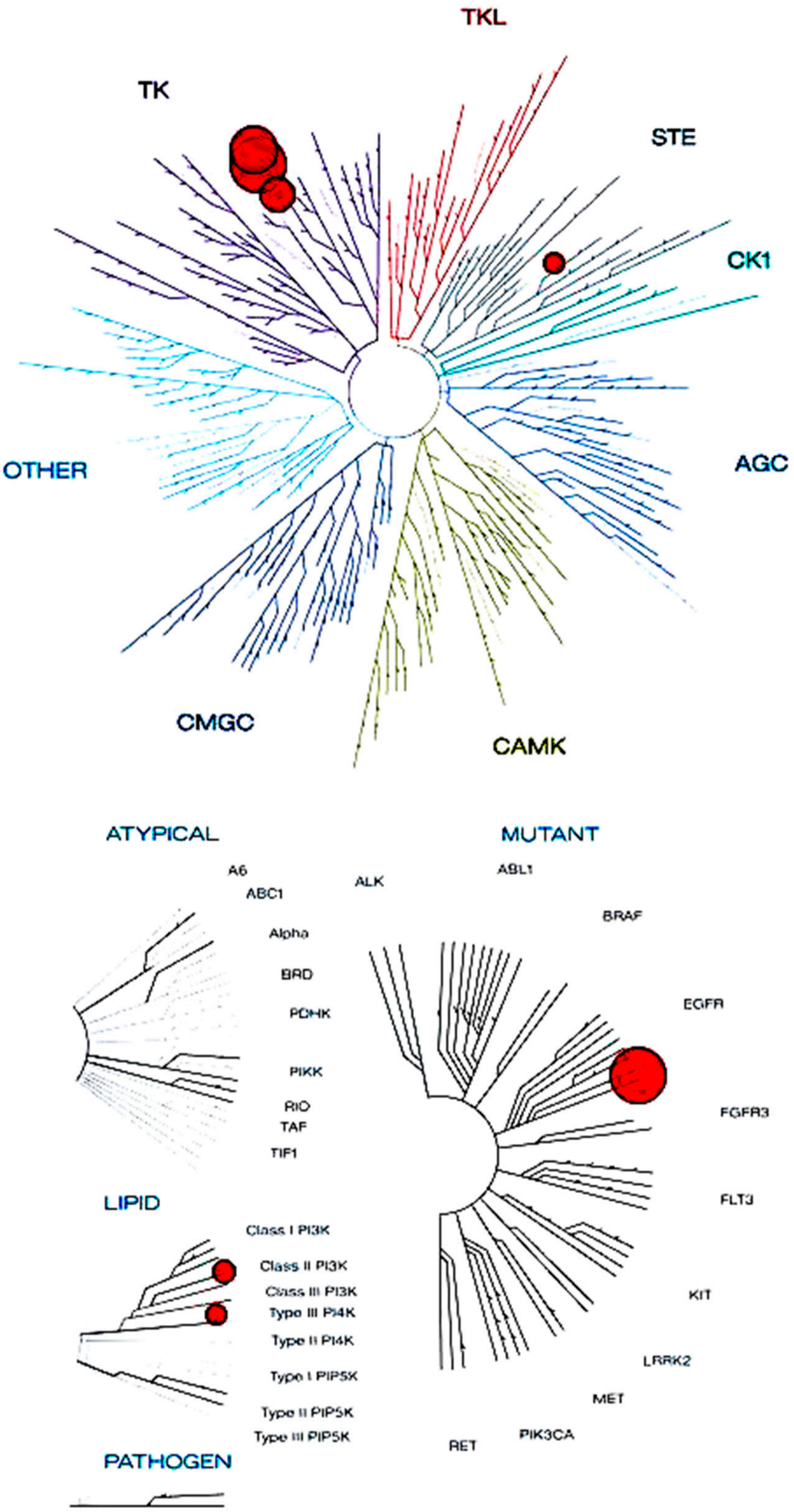

Figure S2. Profile of **Lap** against 468 protein kinases. Relative binding affinities are indicted by red circles in a phylogenetic kinome tree for wild-type enzymes and atypical/mutant/lipo/pathogen variants.. Image generated using TREEspot™ Software Tool (KINOMEscan®, a division of DiscoveRx Corporation,© DISCOVERX CORPORATION 2010).
